# Experience of a tertiary acute care hospital in Southeast Asia in initiating patient engagement with the aid of digital solutions

**DOI:** 10.3389/frhs.2024.1416386

**Published:** 2024-10-01

**Authors:** Peijin Esther Monica Fan, Shu Hui Lim, Guan Hua Jonathan Sim, Mary Jane Seville Poticar, Wee Fang Kam, Yee Fenn Rena Leong, Xin Yi Selene Choy, Lay Teng Ong, Xia Wang, Soy Soy Lau, Gaik Nai Ng, Tracy Carol Ayre, Shin Yuh Ang

**Affiliations:** ^1^ Nursing Division, Singapore General Hospital, Singapore, Singapore; ^2^ Nursing Administration, National Heart Centre Singapore, Singapore, Singapore; ^3^ Division of Nursing, KK Women’s and Children’s Hospital, Singapore, Singapore; ^4^ Nursing Division, Changi General Hospital, Singapore, Singapore; ^5^ Group Nursing, SingHealth, Singapore, Singapore

**Keywords:** patient engagement, technology, patient education, implementation, digital solutions

## Abstract

**Introduction:**

With the goal of patient engagement, an initiative was formulated to equip each patient in the general wards with a tablet whereby they can access their health information and patient education materials and communicate with their healthcare team. This paper presented the methodology of the implementation efforts as well as an evaluation of the preliminary outcomes.

**Methods:**

The process of hospital-wide implementation was shared using the implementation research logic model. The bedside tablets were rolled out hospital-wide in a step-wedge manner over 12 months. Barriers and facilitators to this implementation were discussed together with strategies to optimize the situation. Preliminary outcomes of the implementation were evaluated using the RE-AIM framework.

**Results:**

The initial adoption rate for the bedside tablet was low. Additional strategies, such as survey audits and provision of feedback, development of education materials for patients, facilitation, and purposefully re-examining the implementation strategies, were used to improve adoption. The trend of adoption increased over the course of 2 years from the start of implementation.

**Discussion:**

The initial lower adoption rates may reflect Singapore's paternalistic healthcare culture. While this implementation was driven by the need to move away from paternalism and toward patient engagement, more time is required for significant cultural change.

## 1 Introduction

A focus on patient engagement emerged over the years (1), favoring patient-centered care over paternalism. This was driven by the belief that it is the patient's right to be involved in their care (2), as well as benefits that stem from patient engagement (2, 3).

Although there is currently no standard definition of patient engagement, the concept analysis of 96 articles performed by Higgins et al. (4) provides a comprehensive overview of it. According to Higgins et al. (4), patient engagement may be seen as providing patients with what they need for them to actively participate in their care. The four main attributes of patient engagement are as follows: (1) personalization of interventions or strategies according to the individual needs of the patient; (2) ability and confidence of patients to obtain necessary resources; (3) commitment (willingness) of the patient; and (4) therapeutic alliance, an effective relationship between patients and healthcare staff that supports the pursuit of health goals.

The benefits of patient engagement have been reported in the literature. They range widely from better disease control, medication adherence, and reduced healthcare costs (2) to mortality reductions, improved quality of care, lower medical error rates, and greater satisfaction in the experience of care (3). Hence, patient engagement is an approach to attain the “triple aim” of better health outcomes, improved patient care, and reduced costs (5). As such, major healthcare providers, government agencies, and patient advocates are working to promote and enhance patient engagement (2).

This paper provides a descriptive summary of a tertiary hospital's efforts to initiate patient engagement with the aid of digital solutions. The summary includes illustration of the strategies adopted, barriers, and facilitators of the implementation efforts as well as evaluation of outputs and outcomes.

## 2 Background

Singapore General Hospital is the largest acute tertiary hospital in Singapore, with over 50 specialties within the campus (6). In the Asian context, the paternalistic culture promotes the mindset where many deem their health best left in the hands of doctors who have undergone years of medical training (7). With the goal of moving away from this and promoting patient engagement, the team worked toward making patient's own health records, care plan, and patient education materials more readily available during their hospital stay.

An initiative was formulated to equip each patient in the general wards with a tablet whereby they can access their health information (e.g., trend of vital signs, trend of laboratory results, medication list, upcoming laboratory and radiology orders, dietary restrictions, schedule, and more) and patient education materials, and communicate with their healthcare team. Features in the bedside tablet will be discussed in greater detail in the implementation section of this paper.

This initiative was aligned with the Singaporean government’s direction to transform Singapore into a Smart Nation where we harness the benefits of technology to improve how we live, work, and play (8).

## 3 Implementation methodology

**Figure 1:**
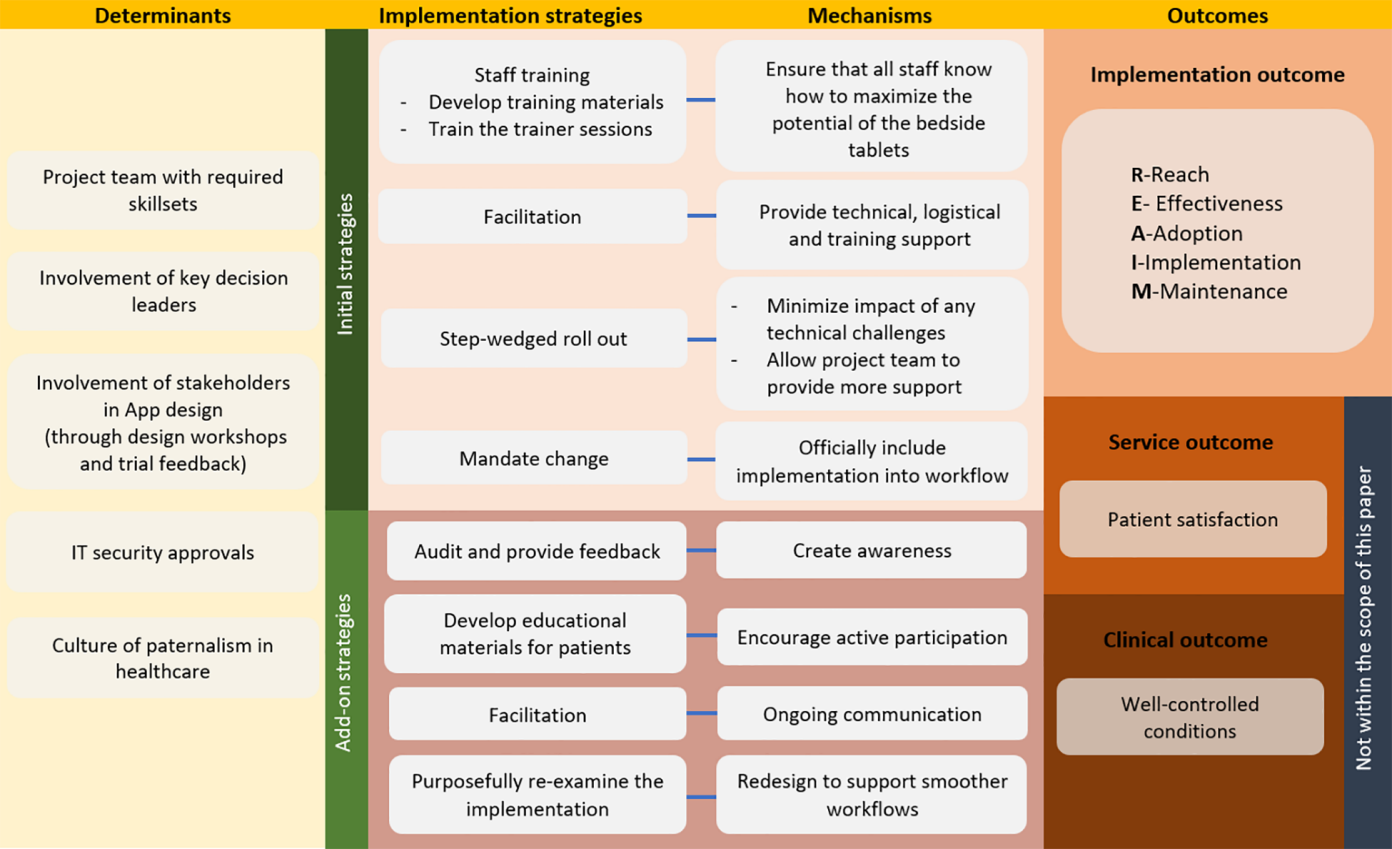
Overview of the implementation of the patient bedside tablet using the implementation research logic model (IRLM).

The implementation of the patient bedside tablet is discussed according to the implementation research logic model (IRLM) (determinants, implementation strategies, mechanisms, and outcomes). Figure 1 shows an overview of the implementation.

### 3.1 Determinants

Determinants are factors that either negatively (barriers) or positively (facilitators) impact an implementation (9).

#### 3.1.1 Project team with required skillsets

The project team consisted of nurses, mobile application (app) developers as well as colleagues from Synapxe, the IT arm of the healthcare system in Singapore.

Nurses in the team provided the app developers with inputs on user requirements and acted as facilitators who were heavily involved in the design, implementation, and evaluation of the initiative. They also provided local technical assistance where possible and sought help from IT colleagues if more technically in-depth troubleshooting was required. Nurses in the team also identified early adopters in each area where the bedside tablet will be launched and conducted training on how to utilize the bedside tablet using train-the-trainer strategies. Involving Synapxe colleagues was necessary as they provided advice and sought approvals pertaining to IT security issues.

#### 3.1.2 Involvement of key decision leaders

Planning for the bedside tablet initiative started in 2018. Senior management support was sought before the start of the project. With senior management's buy-in, it guarantees that required resources for testing and implementation of the initiative will be available to the project team (10). Senior management can also fuel change by advocating for the new initiative and reinforcing the project's vision throughout the organization (10).

#### 3.1.3 Involvement of stakeholders in app design (nurses and patients)

The design of MyCare App took place in several phases (Table 1 shows an overview of functions of the applications in the bedside tablet through the course of app design). Working according to the AGILE methodology, which is based on iterative and incremental development, after each launch of a new version, evaluation and refinement of the solution are carried out before the launch of the next version (11).

**Table 1:** Overview of functions of the applications in the bedside tablet through the course of app design.

Functions	MyCare App v1.0	MyCare App v1.1	MyCare App v2.0 MyEdu App v1.0 NurseHub App v1.0
My Schedule tab	• View a rough daily schedule for the week (mealtimes, doctors’ rounds, and medication times) • View pending radiological and laboratory investigation orders	• View a rough daily schedule for the week (mealtimes, doctors’ rounds, and medication times) • View pending radiological and laboratory investigation orders	• View a rough daily schedule for the day^a^ (mealtimes, doctors’ rounds, and medication times) • View pending radiological and laboratory investigation orders
Feedback for improvement		• Schedule changes in the acute care setting occur too frequently and a weekly schedule may confuse the patient as what they view in the schedule 3 days from now may no longer be the plan when the time comes	
My Care tab	• View vital signs (latest as well as a trend of all vital signs taken during that admission) • View limited blood results (only renal panel, full blood count, and a few other results were shown) • View medication list	• View vital signs (latest as well as a trend of all vital signs taken during that admission) • View limited blood results (only renal panel, full blood count, and a few other results were shown) • View medication list, including chemotherapy orders^a^	• View vital signs (latest as well as a trend of all vital signs taken during that admission) • View all laboratory results except those that may be more sensitive (e.g., HIV and histopathology results)^a^ • View medication list, including chemotherapy orders
Feedback for improvement	• Show chemotherapy orders • Show all blood results rather than a select few		
My Requests tab	• Select specific basic requests within the app	• Requests shown as widgets (without having to enter the app) to reduce clicks required to raise a request^a^	• Requests shown as widgets (without having to enter the app) to reduce clicks required to raise a request • NurseHub was designed to be a nurse-fronting app installed on a smart phone^a^ • NurseHub allows nurses to receive alerts to requests sent by patients via MyCare App^a^
Feedback for improvement	• Make the patient requests tab more accessible	• Allow staff to be alerted to patient's requests raised in MyCare App while they are on the go	
My Profile tab	• View current bed location • View name and contact number of main caregiver recorded in the system • View selected patient education materials • View patient orientation materials in PDF	• View current bed location • View name and contact number of main caregiver recorded in the system • View selected patient education materials • Patient orientation videos were provided for all compulsory patient orientation topics. These videos were in English but with English, Mandarin, Malay, and Tamil subtitles^a^	• View current bed location • View name and contact number of main caregiver recorded in the system • View selected patient education materials • Patient orientation videos were provided for all compulsory patient orientation topics. These videos were in English but with English, Mandarin, Malay, and Tamil subtitles • All patient education materials, whether in PDF or video format, are made available to the patients in a separate MyEdu App^a^ • NurseHub allows nurses to select patient education materials specific to patient needs and recommend these readings to the patient. These materials will then appear to patients in MyEdu under a section “Recommended for you”^a^
Feedback for improvement	• Provide patient orientation videos rather than PDF documents	• Allow easy access to an entire range of patient education materials • Allow staff to curate patient education materials for specific patients	
My Care Team tab	• View name of consultant in charge of the patient's care • Send and receive non-urgent messages to/from the care team	• View name of consultant in charge of the patient's care • Send and receive non-urgent messages to/from the care team	• View name of consultant in charge of the patient's care • Send and receive non-urgent messages to/from the care team • NurseHub allows nurses to receive alerts to messages sent by patients via MyCare App^a^
Feedback for improvement		• Allow staff to be alerted to patient's questions raised in MyCare App while they are on the go.	

^a^ Changes made in the app based on feedback from previous versions.

Nurses, the group of healthcare workers who have the closest contact with patients during their inpatient admission, were included in design workshops to brainstorm ideas for functions of the mobile app. Ideas of patient needs gathered from these workshops were then sorted to determine which were feasible to be included in the first version of the app (refer to Table 1 for the key functionalities of MyCare App through the app design phases). Once the app was designed and IT security approvals sought, version 1.0 of MyCare App was developed.

The first version of MyCare App, version 1.0, was rolled out for feedback in November 2018 in a small hematology ward. This ward was identified to be an early adopter due to the patient profile being that of a younger patient group who were more likely to be technology savvy and were known to be eager to keep track of their blood results and vital signs. The project team chose to target a group most likely to utilize the bedside tablet to maximize the feedback gathered from the first pilot. Qualitative feedback from patients and staff provided us with useful insights that we used in the design of the next iteration (Table 2). Solution fidelity was also evaluated to correct any incorrect functioning as well as to improve the user interface if required.

**Table 2:** Feedback on MyCare App version 1.0 and refinements made.

• Feedback	• Refinements
- Patients were generally satisfied with MyCare App, especially with the ability to view real time updates of their blood results. However, they requested for all blood results to be shown there, rather than only the selected few.	- The team started the process of seeking approval from medical board to show all blood results.
- Patients requested for chemotherapy orders to be shown together with the medication list.	- Chemotherapy orders were shown in MyCare App v1.1.
- Patients preferred to call for the nurse using the call bell at the bedside, or to wave to a nurse in the vicinity rather than to perform multiple steps to reach for the tablet at the bedside and to enter MyCare App, reach the correct tab, and to click on the item required. This was likely so as majority of the rooms in the hospital were multi-bedded rooms where it was easy to locate and call for a nurse.	- Requests were shown as widgets to reduce clicks required to raise a request.
- Patients suggested for MyCare App to be in multiple languages.	- To attempt to heed to that request, the team changed the language via the settings in the bedside tablet. Although the words within MyCare App were translated, information pulled from the EMR remained in English. As it was not possible to change the language of the EMR, the team did not prioritize this suggestion.
- Staff had feedback for orientation materials to be more engaging, in a video format akin to safety videos on a plane ride, rather than in PDF.	- Patient orientation videos were provided for all compulsory patient orientation topics. These videos were in English but with English, Mandarin, Malay, and Tamil subtitles.
- Both staff and patients highlighted that the protective cover for the bedside tablet is very heavy.	- The team sourced for protective covers of a lighter weight that still met the infection control recommendations of the hospital.
- Patients gave feedback that the cord securing the bedside tablet to the bedside locker was too short making it difficult to use the tablet while resting in bed.	- The team sourced for longer cords for securement for future roll-out.
- One technical challenge faced during this trial was that MyCare App was “untrusted” at irregular time intervals and had to be “retrusted” every 2–4 days (in layman terms, this means that the patient could not access their information after every 2–4 days). In the intended functioning of MyCare App, the nurse will use the app to scan the patient's identification barcode upon patient admission to launch this app. After scanning, MyCare App is expected to work throughout the patient's inpatient stay. However, during this first trial, the nurse had to keep rescanning the patient's barcode every 2–4 days.	- This issue was resolved by the app developers before the launch of MyCare App v1.1.

After refinements were made based on the feedback on MyCare App version 1, MyCare App version 1.1 was launched for further feedback gathering in the hematology ward as well as in a medical ward. The medical ward was selected to trial the solution in a different patient group (likely patients who were elderly and with a shorter length of stay compared to the hematology ward). This medical ward was also the designated “smart ward” of the hospital where new initiatives are test bedded. Again, the team gathered qualitative feedback from patients and staff to inform the refinement of the app (Table 3).

**Table 3:** Feedback on MyCare App version 1.1 and refinements made.

• Feedback	• Refinements
- Patients continued to request for all blood results to be shown there, rather than only the selected few.	- The team sought approval from the medical board to display all laboratory results except those that may be more sensitive or require additional explanations by the healthcare team (e.g., HIV and histopathology results).
- Patients found it confusing when the documented schedule for subsequent days changes.	- As the plan for an acutely ill patient might change constantly, it is inevitable that the schedule for the next few days will remain fluid. Hence the team decided to only show the schedule one day at a time, and only the schedule of the current day is shown.
- Due to security concerns, MyCare App will require a password to unlock after being idle for a preset duration. The password to unlock MyCare App is a default password personalized to the individual patient. Staff raised concerns that some patients may wish to set their own password to unlock the app and although this is currently allowed, the user interface is not intuitive for the users.	- MyCare App was redesigned to feature a pop-up prompt during the first scan in of the patient. The prompt asks patients if they wish to set their own passcode instead of using the default one. If they wish to set their own passcode, it may be done at the click of a button.
- Nurses felt that it was additional work to manually reset MyCare App after each patient's discharge in preparation for the next patient's admission.	- MDM was set up. This allowed all devices to be remotely controlled by IT personnel. - MDM allows software updates to be remotely pushed to the tablets. Some troubleshooting may also be performed remotely by IT staff when a fault is reported. - Using MDM, the tablet is automatically and remotely reset once a patient is discharged from the hospital. This removes the need for the nurse to manually reset the tablet when the patient is discharged.
- Staff suggested that we should include the entire range of patient education materials for the patients rather than a selected group of it.	- All patient education materials whether in PDF or video format are made available to the patients in a separate MyEdu App.
- Staff wanted a way to specially curate patient education materials for specific patients. - Staff requested for a more convenient way to be alerted to patient's requests and questions raised in MyCare App. It is currently viewed via a webpage on a laptop or desktop.	- NurseHub was designed to be a nurse-fronting app installed on a smart phone. - Nurses are able to select patient education materials specific to patient needs and recommend these readings to the patient using NurseHub. These materials will then appear to patients in MyEdu under a section “Recommended for you.” - NurseHub allows nurses to receive alerts to requests or messages sent via MyCare App.
- One technical challenge faced during this trial was discrepancies in the caregiver contact number showing in MyCare App and in the EMR.	- This issue was brought to the attention of IHiS personnel and app developers for their investigation and it was corrected before the next launch.

**Figure 2:**
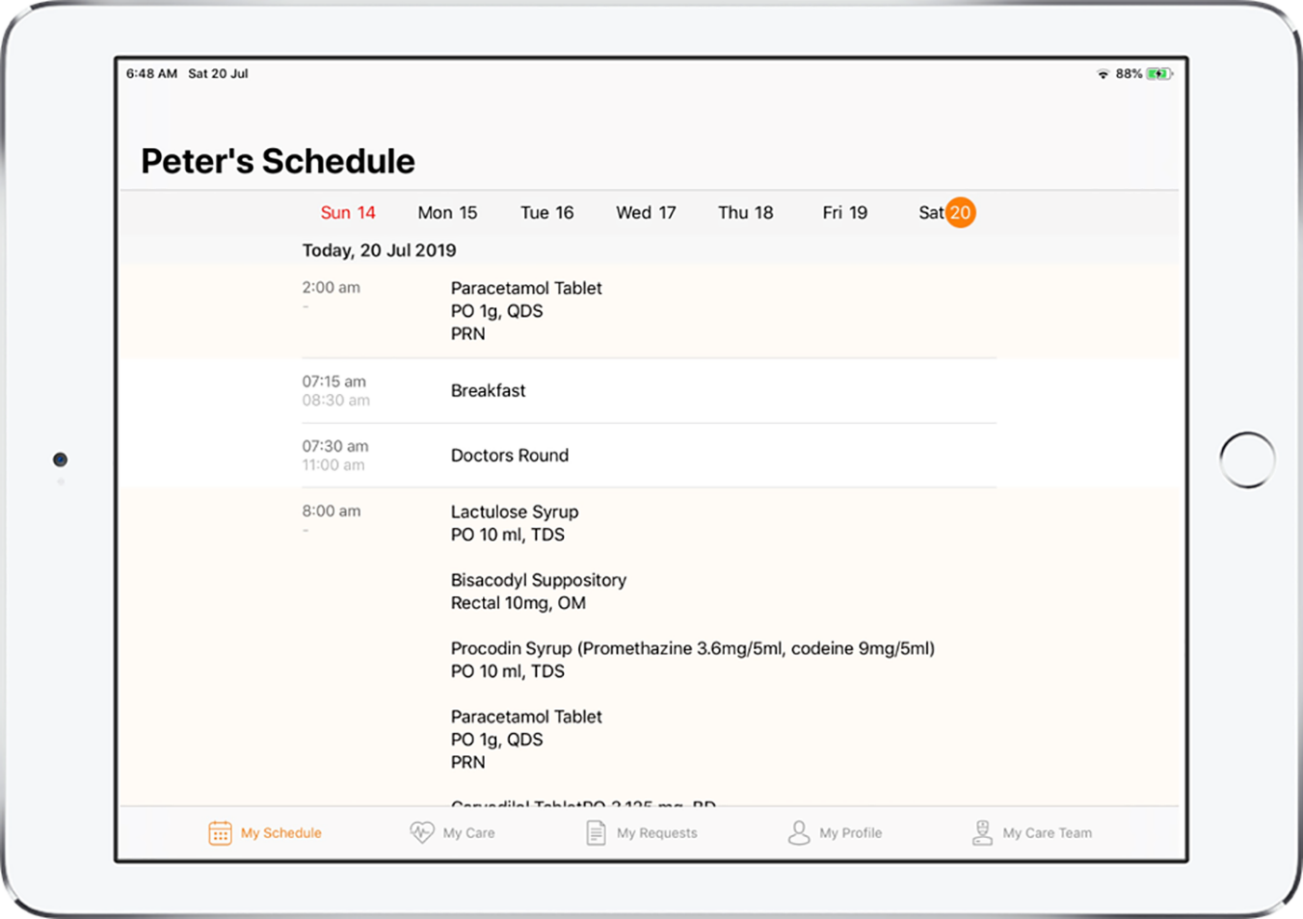
Screenshot of MyCare App displaying the care schedule of the patient.

**Figure 3:**
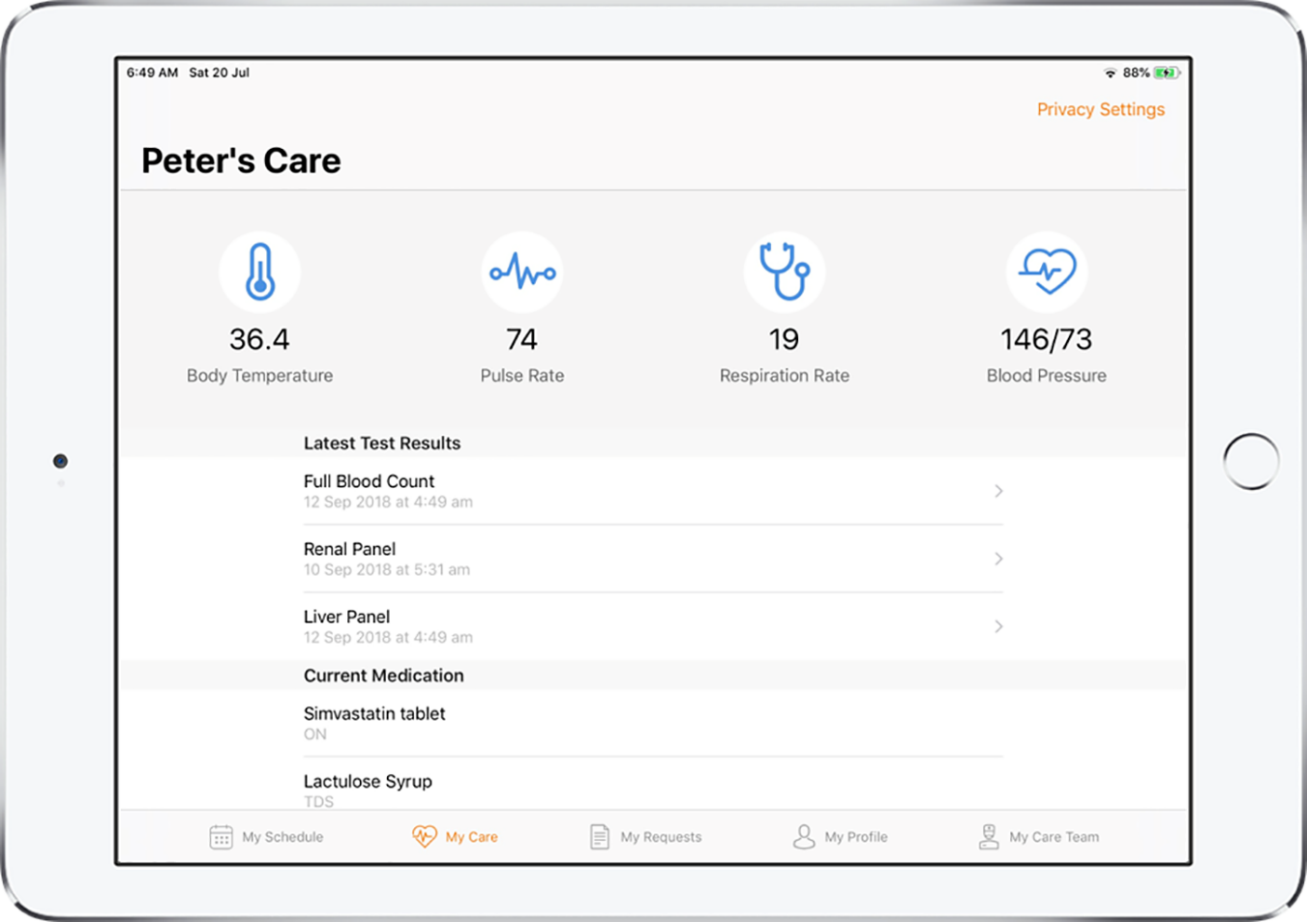
Screenshot of MyCare App displaying the vital signs, laboratory results and current medication list of the patient.

**Figure 4:**
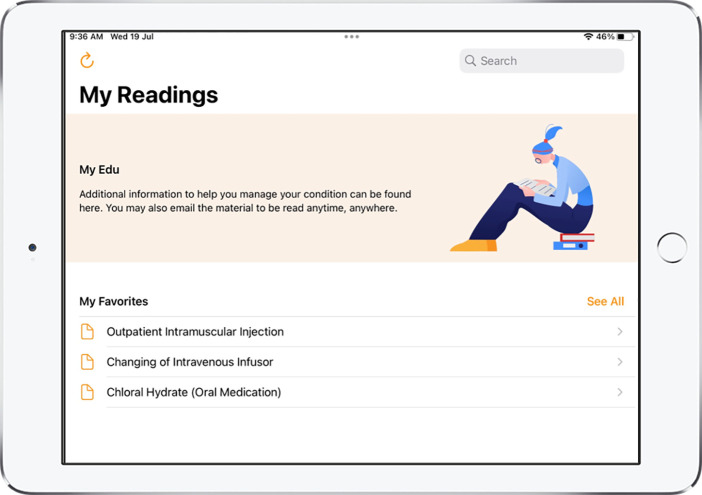
Screenshot of MyEdu App.

**Figure 5:**
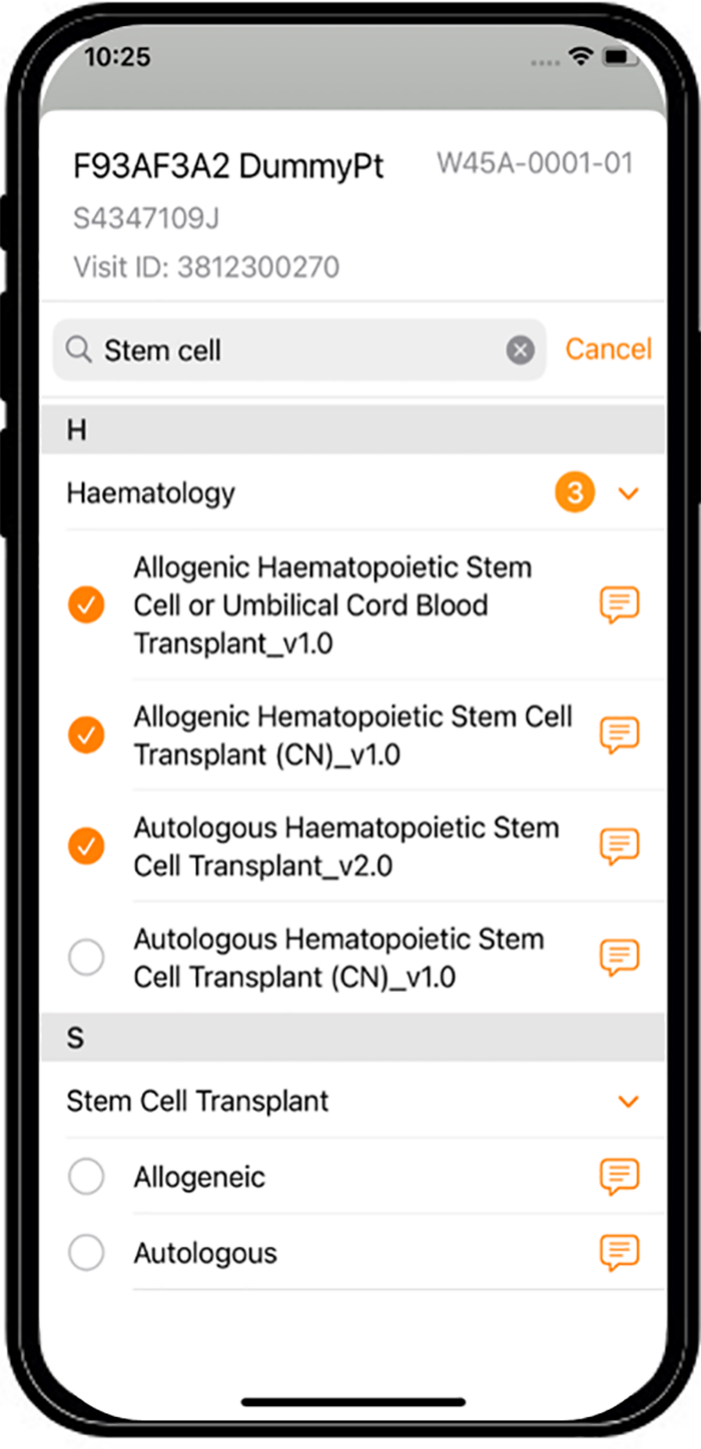
Screenshot of NurseHub App.

MyCare App v2.0, MyEdu App v1.0, and NurseHub App v1.0 were developed as a result of the refinements made and these were the apps utilized for the hospital-wide implementation. Refer to Figures 2–5 for screenshots of these apps.

#### 3.1.4 IT security approvals

As MyCare App was a hospital-wide implementation that required integration to the hospital's electronic medical records (EMR), IT security approvals from the Steering Committee were required. This was crucial to the team and organization as they had oversight on how the implementation would impact on current systems and vice versa (12). The Steering Committee was also able to advise on the steps and activities required for the solution to be implemented and recommend a timeline that considered the various other plans and strategies for the overall IT infrastructure and system that might impact on the solution (12).

#### 3.1.5 Culture of paternalism in healthcare

As mentioned above, promoting patient engagement was one aim of this implementation. The culture of paternalism in healthcare within an Asian context is definitely one of the main barriers that was challenging to manage as it was a cultural mindset beyond the control of the project team (7). As much as the project team and implementation are able to have its influence on culture (coming from a healthcare setting), culture is something that will need a longer time to change.

### 3.2 Implementation and mechanisms

This section presents the implementation strategies used to promote the adoption of the bedside tablet solution into usual care as well as the mechanisms of actions of how these strategies impact on the outcomes (9). Refer to the initial strategies in Figure 1 for an overview.

#### 3.2.1 Staff training

Staff training was essential as this was a completely new solution and training will be required to ensure that all staff acquire the knowledge required for the potential of this solution to be fully realized.

The project team developed a step-by-step guide placed in the staff electronic learning (e-learning) platform. This guide was accessible by all nursing staff and it provided steps for app usage. Staff were encouraged to complete this e-learning module before face-to-face training sessions conducted by the team.

The project team went to individual wards to conduct face-to-face training before roll-out in the wards. As it was not possible to train every single member of staff, the team utilized the train-the-trainer strategy where the project team trained the nurse champions identified by the nurse managers of the ward and these champions conducted training among their fellow ward colleagues.

#### 3.2.2 Facilitation

To cushion the impact of the new implementation on daily busy workflows and to support the nurses in the wards with the major change, the role of the project team as facilitators was emphasized. With each roll-out, the project team went to the wards regularly to troubleshoot problems, provide technical support, and feedback to the IT team when technical challenges were faced. Weekly meetings were held by the project team with the IT helpdesk to examine the problems reported and to attempt to find the root cause. As the project stabilized, the frequency of the meetings decreased.

#### 3.2.3 Step-wedge roll-out

**Figure 6:**
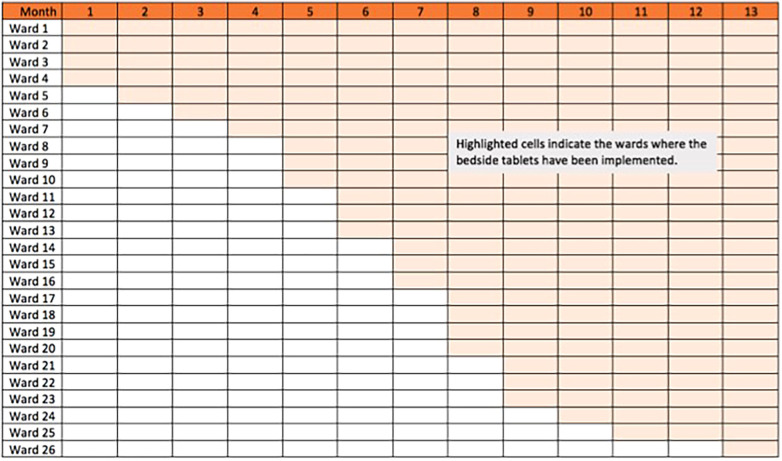
Step-wedge roll out.

Because of the heavy involvement of the project team with each roll-out, and to minimize the impact of any technical bugs that did not surface during the trial period in the first two wards, a step-wedge method was used to deploy the solution where we deployed the solution to a total of 27 general wards within a span of 12 months (Figure 6). This allowed the project team to provide more attention and support to each ward as they underwent the change.

#### 3.2.4 Mandate change

The team formally included the use of the bedside tablet as part of patient and family orientation by including it as a performance standard in the hospital's mass competency assessment for “Patient and Family Education.” This strategy was to officially include this implementation as part of standard practice and ensures that all staff (current and future) will be informed and trained on its use.

### 3.3 Outcomes

Outcomes for implementing an initiative may be categorized as implementation outcomes (success of the inclusion of the new initiative to usual practice), service outcomes (satisfaction of patients), and clinical outcomes (health-related outcomes). For this paper, we will focus on the implementation outcomes.

The RE-AIM framework was used to evaluate the implementation outcomes of the bedside tablet implementation. RE-AIM is a planning and evaluation model that addresses five dimensions of individual- and setting-level outcomes important to program impact and sustainability: Reach, Effectiveness, Adoption, Implementation, and Maintenance (13).

#### 3.3.1 Reach

Reach is the percentage of eligible participants who were included and excluded, and how representative of the population they were (14). For this project, 100% of general ward patients received the bedside tablet solution. Those excluded were patients who were admitted in high-dependency or intensive care units who were unlikely to be well enough to utilize the functions in the tablets. Hence, the project performed well in this aspect.

#### 3.3.2 Effectiveness

Effectiveness is defined as the impact of the intervention on those who have used the initiative (14). We measured this using the informal qualitative feedback obtained from patients.

We received some positive feedback from patients who provided feedback that it was easy for them to check their upcoming schedule and results without having to ask a nurse, and some found the medication list helpful. Patients also felt that the education materials were useful and enabled them to be better informed.

#### 3.3.3 Adoption

Adoption may be understood as the percentage of patients who participated in the initiative (14). The project team saw adoption in two aspects: adoption by staff as well as adoption by patients. The latter was dependent on the former. Staff adoption is defined by the team as the rate of the staff enrolling patients to the bedside tablets upon patient admission, while patient adoption is the rate of patients who utilized the bedside tablet.

**Figure 7:**
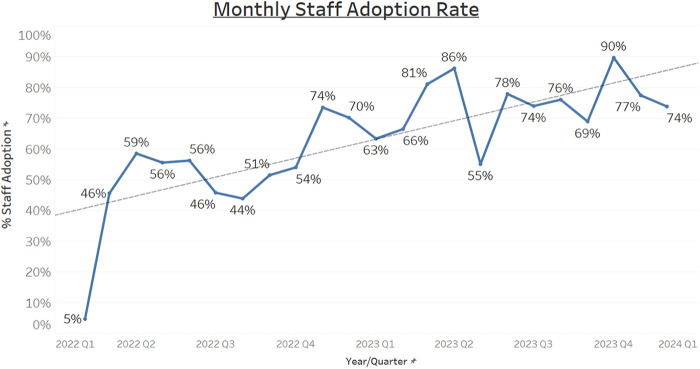
Trend of staff adoption over time.

Within the initial months of implementation, staff adoption was low. It remained at less than 60% within the first 9 months of implementation (Figure 7).

Patient adoption was not actively tracked during this period. Although we were able to track the usage in terms of the number of clicks within MyCare and MyEdu, we were unable to determine if the clicks were from the same patient or from separate unique patients. But patient adoption was likely to be low when staff adoption was low. During informal visits to the wards by the project team, some patients verbalized that they were unaware that the tablet was for their use.

To promote adoption, add-on implementation strategies were taken. An overview of the add-on strategies are shown in Figure 1.

##### 3.3.3.1 Audit and provide feedback

The adoption rates of both staff and patients were shared regularly to the ward managers as feedback to show how well the ward was performing. This was to create awareness of the low adoption rate and to encourage active steps by the staff to adopt the change.

##### 3.3.3.2 Develop education materials for patients

Posters informing patients and caregivers of this initiative were placed in patient rooms, at the ward entrance, and in corridors. The purpose was to encourage its use among patients who had already been enrolled into MyCare App and to increase adoption by staff (as patients or caregivers may approach them to enroll the patient if they were not already enrolled).

A short video to introduce the bedside tablet initiative was created and uploaded to MyEdu App. This allowed patients to learn how to use the app independently.

In addition, a simple guide to the use of the bedside tablet was printed as a sticker and pasted on the patient’s tablet to empower patients and family members to self-help during their stay.

These materials hoped to encourage the patients to be active participants in their care.

##### 3.3.3.3 Facilitation

Ward engagement sessions were conducted by the project team to collect user feedback and share upcoming features. Monthly MyEdu newsletters were also launched to inform nurses of the newly uploaded patient education materials and to encourage them to use this platform to share them.

##### 3.3.3.4 Purposefully re-examine the implementation

One item of feedback for MyCare App v1.1 was that nurses felt it was additional work to manually reset MyCare App after each patient's discharge in preparation for the next patient's admission. In MyCare App v2.0, the tablet was automatically and remotely reset by mobile device management (MDM) once a patient was discharged from the hospital. This allowed all devices to be remotely controlled by IT personnel and removed the need for the nurse to manually reset the tablet when the patient was discharged (Table 3).

However, as MDM was unable to control individual apps, it was not able to reset only MyCare App without resetting the entire tablet to its original factory settings. This led to a new problem of it requiring six steps and a long waiting time (sometimes up to 15 min to connect the tablet to the correct network and for the apps to be loaded into the tablet) before the patient could be enrolled into MyCare App. This was one pain point frequently brought up by staff. The additional steps of connecting the tablet to the dedicated network as well as to scan the patient to set up their account were seen as a chore by nurses.

The team finally decided that the task of manually resetting the app upon discharge was far less tedious than the task of having to set up the tablet from factory settings; therefore, we reverted to not having MDM remotely reset the tablets to factory settings upon patient discharge.

Another major redesign carried out by the project team was enhancing the process of setting up MyCare App from scanning a barcode on the patients’ wrist tag to scanning a QR code. This made it easier to scan from any angle compared to a barcode.

#### 3.3.4 Implementation

Under the RE-AIM framework, “implementation” is the extent to which the initiative was delivered as intended (14). Some technical challenges faced during this project, such as weak WiFi signal strengths in certain areas, led to the user experience being less ideal than intended. Otherwise, the bedside tablet initiative was, to a large extent, delivered as intended.

#### 3.3.5 Maintenance (stickiness)

Maintenance monitors the long-term impact of the study (14). The project team continued to monitor monthly adoption rates. The results showed an increasing trend in adoption over the span of a few months (Figure 7).

There was also growing interest from other healthcare staff in the use of the bedside tablet for other functions. Examples include inpatient video consultations with a doctor, a tool to encourage patients to mobilize outside of therapy, and for pharmacists to perform virtual medication reconciliation sessions.

## 4 Discussion

Providing all patients admitted in Singapore General Hospital's general wards with easily accessible personal health information was implemented with the intention of increasing patient engagement, a factor touted to offer a wide range of benefits, such as improved disease control, medication adherence, increased cost benefits (2), mortality reduction, improved quality of care, lower medical error rates, and greater satisfaction in the experience of care (3).

The initiative aims to promote the attributes of patient engagement in several ways.

### 4.1 Attribute 1: personalization of interventions or strategies according to the individual needs of the patient

This initiative supports this attribute by allowing healthcare professionals to curate education materials specific to the patient and form the first step toward presenting a comprehensive care plan for the patient that is readily available to them. Although the current functions of the available apps are unable to fully achieve this yet, the availability of a tablet by the bedside gives the healthcare professionals the potential to develop future solutions that can provide patients with their consolidated care plan in a convenient manner. The provision of personal health information is central to patient engagement as patients will require these for self-management of their conditions (15).

### 4.2 Attribute 2: ability and confidence of patients to obtain necessary resources

The full library of patient education materials is made easy to access within the bedside tablet. Patients can also email any piece of education material to themselves for future reference.

### 4.3 Attribute 3: commitment (willingness) of the patient

There is an indirect contribution of the bedside tablet initiative to this attribute where the convenience of accessing their health information and education should increase the willingness of patients to use it for health-seeking behaviors, such as monitoring the trend of their blood results.

### 4.4 Attribute 4: therapeutic alliance

The bedside tablet allows for communication between healthcare staff and patients. This hopes to strengthen the therapeutic relationship.

With increasing health literacy among Singaporeans, there was an expectation that patients or their caregivers wanted to be engaged as much as we would like to engage them. Hence, the poor early adoption rates among patients and staff came as a surprise to the project team. However, this was in line with the Diffusion of Innovations theory, where only 16% of the population are innovators and early adopters of change (16). More time is required for the large majority to accept and adopt the change.

Implementation projects are commonly backed by the setting's readiness and appetite for change (17). But unlike the usual process of scaling up only when promising adoption is proven, this was scaled hospital-wide despite low adoption rates at the beginning. The reasons for this are worth discussing as it is a challenge faced when implementing initiatives in a real-world scenario.

One main reason why the initiative was scaled hospital-wide despite low initial adoption rates was because the team, with support of hospital management, recognized the complex interrelations between the elements of adoption, functionality, and funding. Adoption will increase for solutions with higher functionalities, while the building of functions will require funding. Good adoption rates support greater funding, which will, in turn, lead to more functionalities built and an even better adoption rate.

This cycle may be a beneficial self-sustaining cycle for ongoing, well-developed solutions but it poses a challenge for new implementations. It is not realistic for many functions to be built into the new solution at the initial stages as time will be required to iron out any problems that arise from the implementation. Functionality is also dependent on the degree to which the initiative is scaled as it is difficult to obtain further funding to increase functionality if the initiative is only implemented on a limited number of beds. Hence, as the project team was clear about the hospital's priority for patient engagement, the team decided to first implement the bedside tablet hospital-wide, then continue work to build on the functionality, allowing adoption rates to pick up naturally.

Another reason why the project team pushed through without high adoption rates was because this initiative called for a change in culture and we recognize that change requires time. The implementation of bedside tablets was more than just providing an additional service for patients, it was bringing a change in culture for Singaporean society, promoting a shift away from paternalistic healthcare to patient-centered care.

The team believes that part of the initial poor adoption rates by patients or caregivers is related to the lack of “willingness” to be further engaged [one of the four elements of patient engagement according to Higgins et al. (4)]. Similarly, Franklin and Myneni (5) highlighted patients' active involvement in their health and care to be one of the factors promoting optimal health technology use. From Singaporean healthcare staff’s experiences, there are expectations from patients to be cared for by a professional, rather than claiming the responsibility to partake in joint decision making. A focus group discussion among nurses revealed that some patients are reluctant to partake in activities of daily living, such as feeding or cleaning their faces, although they are capable of doing so (18). There also seems to be a sense that this is a service they pay for and they should not need to have to do anything by themselves, almost as if Singaporean society is not ready to claim a greater ownership in their care.

Patient engagement is almost a necessity in today's healthcare climate given the growing trend of an aging population and the correspondingly smaller workforce. Hence, there is a need to persist in this cultural change. For patients to engage, they must be empowered, as they need motivation and the ability to participate in care (19). In addition, the healthcare provider must maximize the potential and the opportunities for patients to engage (19). Therefore, the project team is aware of the need for this cultural change as well as the time, patience, and efforts required.

Furthermore, as we encourage an increase in adoption, there will be more opportunities for increased functionality (higher adoption will justify increased funding support to increase functionalities). An increased functionality will then increase adoption. This is the feedback loop the team foresee; hence, the step-wedged implementation allowed the team to better facilitate each ward during the roll-out and the team continues to gather feedback and work on additional functionalities requested by the end users.

This study is not without limitations. There are other factors that could impact on adoption of a digital solution. Franklin and Myneni (5) found that health applications with the most accessibility and engagement features had higher user ratings. These criteria were evaluated based on a checklist of features, including font enlargement, zoom, appropriate-sized touch components, clear path to next action, and whether the app informs, engages, or partners with the user (5). MyCare, MyEdu, and NurseHub App have not been rated according to such criteria and it is unsure if sub-optimal features had a negative impact on the adoption rate.

## 5 Conclusion

This paper shared the method of implementation of the bedside tablet initiative in Singapore General Hospital as well as learnings from it. The lower adoption rates may reflect Singapore's paternalistic healthcare culture. While this implementation was driven by the need to move away from paternalism and toward patient engagement, more time is required for significant cultural change.

Future research could study the user interface of the app from the perspective of an elderly patient to see if any accessibility and engagement features could be further improved to increase adoption among the elderly. More research could also focus on reasons for the lack of desire from patients or caregivers to be engaged in their health and care despite encouragement from their healthcare providers to do so.

## Data Availability

The datasets presented in this article are not readily available because the data generated for this paper is not available for sharing. Requests to access the datasets should be directed to lim.shu.hui@sgh.com.sg.
